# Role of Endogenous Cortistatin in the Regulation of Ghrelin System Expression at Pancreatic Level under Normal and Obese Conditions

**DOI:** 10.1371/journal.pone.0057834

**Published:** 2013-02-28

**Authors:** Belén Chanclón, Raúl M. Luque, José Córdoba-Chacón, Manuel D. Gahete, Ana I. Pozo-Salas, Justo P. Castaño, Francisco Gracia-Navarro, Antonio J. Martínez-Fuentes

**Affiliations:** Department of Cell Biology, Physiology and Immunology, University of Cordoba and Reina Sofia University Hospital, Instituto Maimónides de Investigación Biomédica de Córdoba (IMIBIC), and CIBER Fisiopatología de la Obesidad y Nutrición, Córdoba, Spain; University of Santiago de Compostela School of Medicine - CIMUS, Spain

## Abstract

Ghrelin-system components [native ghrelin, In1-ghrelin, Ghrelin-O-acyltransferase enzyme (GOAT) and receptors (GHS-Rs)] are expressed in a wide variety of tissues, including the pancreas, where they exert different biological actions including regulation of neuroendocrine secretions, food intake and pancreatic function. The expression of ghrelin system is regulated by metabolic conditions (fasting/obesity) and is associated with the progression of obesity and insulin resistance. Cortistatin (CORT), a neuropeptide able to activate GHS-R, has emerged as an additional link in gut-brain interplay. Indeed, we recently reported that male CORT deficient mice (cort−/−) are insulin-resistant and present a clear dysregulation in the stomach ghrelin-system. The present work was focused at analyzing the expression pattern of ghrelin-system components at pancreas level in cort−/− mice and their control littermates (cort +/+) under low- or high-fat diet. Our data reveal that all the ghrelin-system components are expressed at the mouse pancreatic level, where, interestingly, In1-ghrelin was expressed at higher levels than native-ghrelin. Thus, GOAT mRNA levels were significantly lower in cort−/− mice compared with controls while native ghrelin, In1-ghrelin and GHS-R transcript levels remained unaltered under normal metabolic conditions. Moreover, under obese condition, a significant increase in pancreatic expression of native-ghrelin, In1-ghrelin and GHS-R was observed in obese cort+/+ but not in cort−/− mice. Interestingly, insulin expression and release was elevated in obese cort+/+, while these changes were not observed in obese cort−/− mice. Altogether, our results indicate that the ghrelin-system expression is clearly regulated in the pancreas of cort+/+ and cort −/− under normal and/or obesity conditions suggesting that this system may play relevant roles in the endocrine pancreas. Most importantly, our data demonstrate, for the first time, that endogenous CORT is essential for the obesity-induced changes in insulin expression/secretion observed in mice, suggesting that CORT is a key regulatory component of the pancreatic function.

## Introduction

Ghrelin gene (GHRL) is a complex gene whose processing generates, either through alternative splicing or post-translational modifications, a wide variety of transcripts and proteins with multiple functions [1], [2], [3], [4]. The main transcript of GHRL (pre-pro-ghrelin) encodes the well-known native-ghrelin (henceforth referred to as ghrelin) and obestatin (23aa). Ghrelin is a 28-amino acid multifunctional hormone predominantly produced by the stomach, but also lower amounts are generated in wide variety of tissues including the pancreas, where it can act as a paracrine/autocrine factor [5], [6], [7], [8], [9]. Ghrelin displays the unique distinctive feature to be acylated at its third residue (Ser) by the addition of a middle-chain fatty acid (n-octanoic acid) catalyzed by the enzyme ghrelin O-acyltransferase (GOAT). Afterward, either acylated or unacylated-proghrelin can be further processed by the prohormone convertase 1/3 (PC1/3) thus generating the acylated-ghrelin or its unacylated-ghrelin counterpart, a form of ghrelin initially considered as inactive ([2]). Ghrelin acylation is crucial for its biological activity, and endows ghrelin as the natural ligand of the ghrelin receptor [formerly known as growth hormone (GH) secretagogue receptor (GHSR)] [10], [11], [12]. In addition to pre-pro-ghrelin, other splice variants of GHRL, such as In1-ghrelin or des-Glu14-Ghrelin can also be generated in several tissues ([13]; [2]). Particularly, the In1-ghrelin variant, which retains the GHRL intron-1 sequence [13], [14], are regulated in a tissue-dependent manner by metabolic status and display patho-physiological relevance at different levels [13], [14]. Importantly, In1-ghrelin variant is also susceptible to be acylated since it shares the same start codon, the signal peptide and the first 12aa of ghrelin, which includes the putative acylation site at Ser3 and the residues found to be necessary for acylation (Gly1 and Phe4) [14].

Although it has been documented that ghrelin-derived peptides/GOAT/GHSR comprise an important regulatory system for the modulation of pancreatic function, questions remain unanswered regarding the mechanism(s) by which the ghrelin-system is locally produced and regulated under normal and obese conditions. In line with this, our group and others have suggested the existence of a unique functional interaction between the ghrelin-system and cortistatin (CORT), a peptide that shares high structural and functional similarities with somatostatin (SST). Specifically, it has been reported that CORT, but not SST, binds with high affinity to the GHSR, and that some endocrine actions of CORT are mediated through the GHSR [15], [16]. Moreover, we have recently reported that endogenous CORT is substantially involved in the control of insulin/glucose homeostasis, which was associated with drastic changes in circulating ghrelin [16]. Based on these observations, the study of the plausible involvement of CORT in the regulation of the different components of the ghrelin-system could shed new light in the complex relationship between these regulatory systems (CORT/ghrelin) in pancreatic function under normal and pathological states. To this end, in the present work we sought to investigate, for the first time, the expression and regulation of the ghrelin–system (native-ghrelin, In1-ghrelin, GHSR and GOAT) as well as of insulin levels in the pancreas of cort+/+ and cort−/− mice under basal and obese conditions.

## Materials and Methods

### Ethics Statement

All experimental procedures for animal care and experimentation were approved by the Ethical Committee of the Cordoba University.

### Animals

CORT knockout mice (cort −/−) and their respective littermate controls (cort +/+) were generated from an in-house breeding colony [16]. Genotypes were determined by conventional PCR from snipped tails as previously described [16]. All animals were housed under standard conditions of light cycle (12 h light: 12 h dark) and temperature (22–24°C), with standard food (SAFE-diets; Barcelona, Spain) and water *ad libitum* until the experimental diet-induced obesity was performed (see below). In order to reduce the stress, animals were handled every day for 2 weeks (wk) before the sacrifice.

### Experimental Design and Diet-induced Obesity (DIO)

At 4 wk of age, male mice were single-housed and fed for 16 wk a low-fat diet (LFD: 10 kcal% from fat, 70 kcal% from carbohydrates and 20 kcal% from proteins) or under a high-fat diet (HFD: 60 kcal% from fat, 20 kcal% from carbohydrates, 20 kcal% from proteins) (Research Diets, Gentofte, Denmark) [17].

### Samples

At the end of the experiment, animals were sacrificed, all efforts were made to minimize suffering, and the pancreas immediately removed and processed for either islet isolation (see below) or RNA isolation. For RNA isolation, whole pancreas was immersed in Trizol reagent, homogenized by a basic ultra-turrax (IKA, Staufen, Germany), snap-frozen in liquid nitrogen and subsequently stored at −80°C until their analysis as described below.

### Islet Isolation and Culture

For islet isolation, whole pancreas was placed in ice-cold collagenase V solution (1 mg/ml) and minced into small pieces. Then, tissue was maintained in a collagenase solution at 37°C for 30–45 min period in a water-bath, and every 10 min pancreatic pieces were mixed by smooth pipetting. After that, pancreatic pieces were treated with DNAse for 10 min at 37°C and washed 3 times with Hank´s solution by 800 rpm centrifugation at 4°C for 30 s. Finally, islets were poured onto a 70 µm cell strainer in order to purify them and cultured in complete RPMI 1640 medium as elsewhere described [18]. Finally, total RNA was isolated from cultured islets.

### RNA Isolation and Reverse Transcription (RT)

Total RNA was isolated according to Trizol reagent manufactureŕs instructions (Invitrogen, Barcelona, Spain) and treated with DNAse kit (Promega, Madrid, Spain). For RNA isolation from pancreatic islets, Absolutely RNA RT-PCR Miniprep Kit with Deosyribonuclease treatment (Stratagene, La Jolla (CA), USA) was used by following manufactureŕs instructions. In both cases, total RNA was quantified by nanodrop and afterward 2 ug of total RNA was reverse transcribed by the RevertAid First Strand cDNA Synthesis Kit (Fermentas, Hanover, USA).

### Quantitative Real-time PCR

PCR conditions, primer design and validation, as well as PCR transcript quantification was performed as previously described [16], [17], [19]. mRNA levels from whole pancreas were normalized by a normalization factor obtained from the quantification of 3 different housekeeping genes [Cyclophilin-A, β-actin and glyceraldehyde-3-phosphate-dehydrogenase (GAPDH)] and generated by the GeNorm 3.3 software [20]. Due to the limited amount of pancreatic islet sample obtained, transcript normalization was performed by adjusting copy number of each transcript by the copy number of β-actin.

### Assessment of Plasma Insulin

Trunk blood was collected from cort +/+ and −/− mice, immediately mixed with MiniProtease inhibitor (Roche; Barcelona, Spain), placed on ice, centrifuged and plasma was stored at −80C until insulin determination. Circulating insulin level was assessed by using a commercial ELISA kit (Millipore; Madrid, Spain).

### Statistical Analysis

Samples from all groups within an experiment were processed at the same time. All data are expressed as mean ± SEM and obtained from a minimum of 3 and a maximum of 7 animals. Statistical differences were determined by using Graph Pad Prism (v.5.0.) software (GraphPad Software, Inc., La Jolla (CA) USA). The effect of obesity and/or CORT deficiency on transcript expression profile and circulating insulin levels in cort+/+ and cort −/− mice was assessed by the Student’s t-test and by ANOVA, followed by Fisheŕs post-hoc test for multiple comparisons. P<0,05 was considered significant.

## Results

### Ghrelin System Expression in Whole Pancreas and Islets from Lean and Obese Cort+/+ and Cort −/− Mice

**Figure 1:**
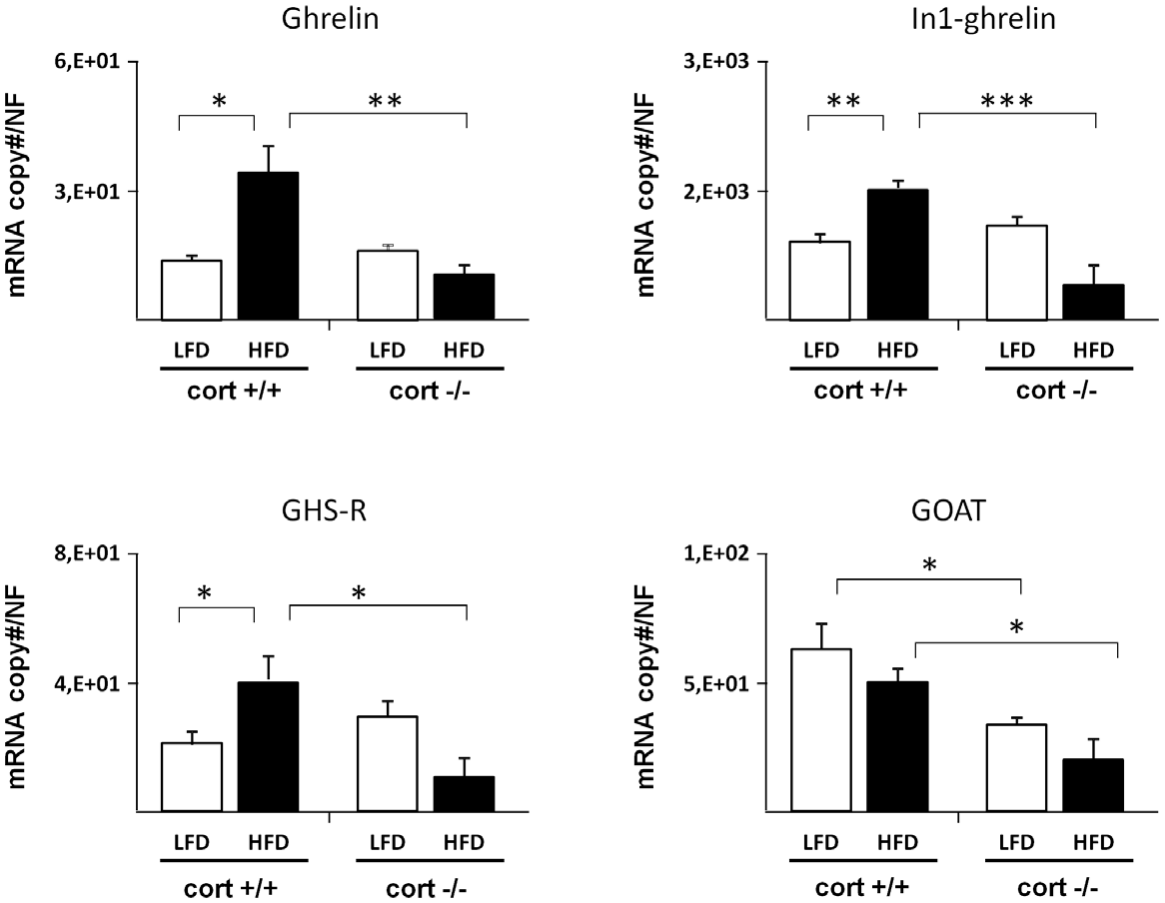
Expression level of different ghrelin system components in the whole pancreas of cort+/+ and cort−/− male mice fed a low fat (LF) or high fat diet (HFD). Values represent mean ± SEM of copy number adjusted by normalization factor (NF) per 100 ng of cDNA obtained from 3–7 animals per experimental group. (*: p<0,05; **: p<0,01; ***: p<0,001).

Expression of ghrelin, In1-ghrelin variant, GHS-R and GOAT in the pancreas of cort+/+ and cort−/− mice were determined by qRT-PCR (Fig. 1). All components of the ghrelin system were expressed in pancreatic extracts of cort+/+ and cort−/− mice; however, their relative expression levels markedly differed. Interestingly, In1-ghrelin expression was substantially higher than that of ghrelin in cort+/+ mice (834.5±92.48 vs. 14.4±1.21 mRNA copies/NF per 100 ng total RNA, respectively; n = 5) as well as in cort−/− mice (1047.6±105.42 vs. 16.0±2.09 mRNA copies/NF per 100 ng total RNA, respectively; n = 4) (Fig. 1). Moreover, GOAT was highly expressed as compared to ghrelin (62.2±9.98 mRNA copies/NF per 100 ng total RNA), and GHS-R showed low expression levels in cort +/+ mice under LFD (21.1±3.65 mRNA copies/NF per 100 ng total RNA) (Fig. 1). Lack of CORT, did not alter the expression of neither ghrelin forms nor GHS-R but significantly decreased GOAT expression (32.3±3.53 mRNA copies/NF per 100 ng total RNA) (Fig. 1).

**Figure 2:**
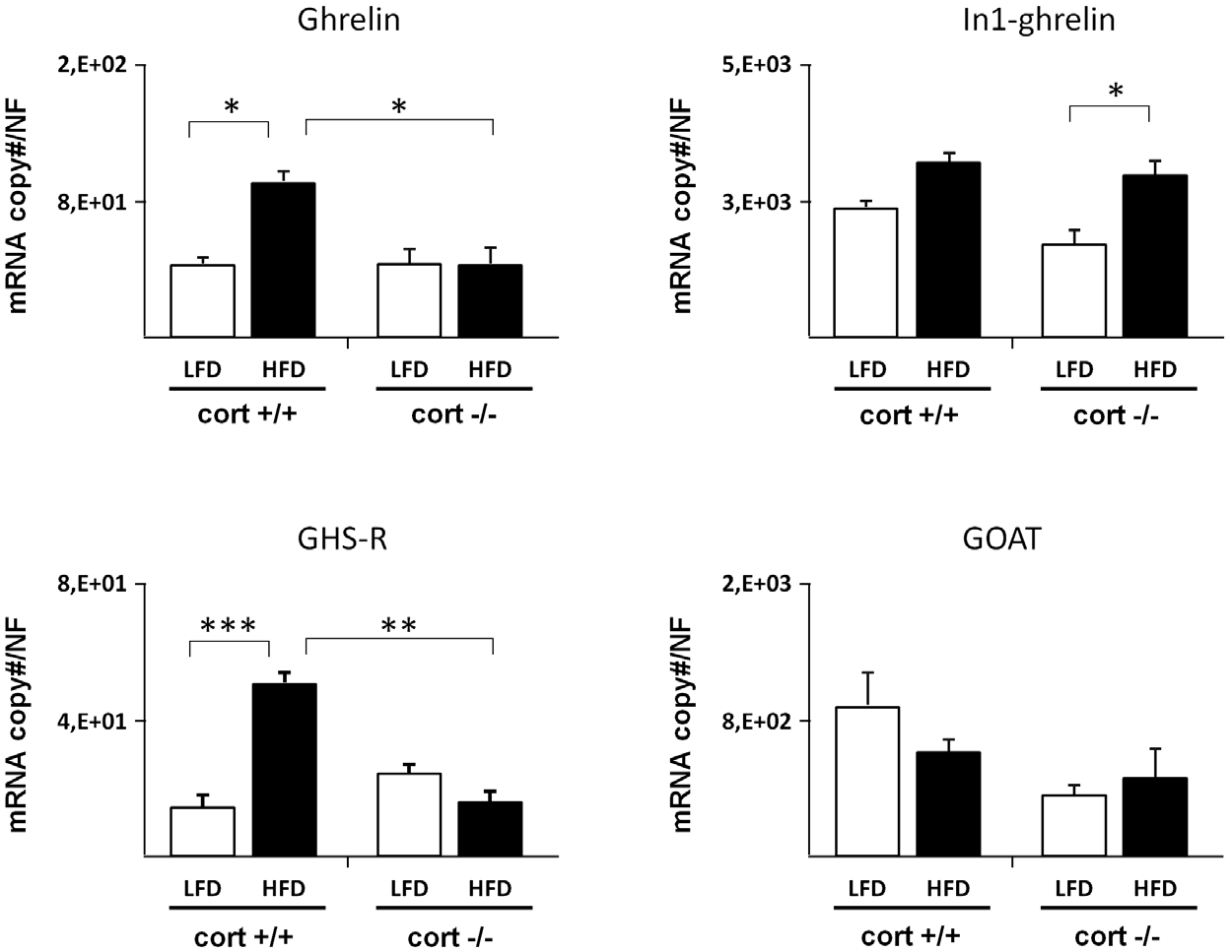
Expression level of different ghrelin system components in pancreatic islets of cort+/+ and cort−/− male mice fed a low fat (LF) or high fat diet (HFD). Values represent mean ± SEM of copy number adjusted by normalization factor (NF) per 100 ng of cDNA obtained from 4–7 animals per experimental group. (*:p<0,05; **: p<0,01; ***: p<0,001).

Administration of HFD to prepuberal animals (4 wk of age) during a 16 wk period caused a dysregulation of some of the components of the ghrelin system in both whole pancreas and islet-enriched fraction of cort+/+ animals (black columns in Fig. 1 and 2). In particular, ghrelin, In1-ghrelin and GHS-R transcript levels were significantly higher in the whole pancreas of cort+/+ animals fed a HFD, while GOAT mRNA levels remained unchanged (Fig. 1). Similarly, ghrelin and GHS-R transcript expression was increased in islet-enriched fraction under HFD conditions, while In1-ghrelin and GOAT were not altered (black columns in Fig. 2).

Absence of CORT not only prevented the obesity-induced increase in ghrelin, In1-ghrelin and GHS-R transcripts but also caused a significant reduction in the expression of these transcripts (black columns in Fig. 1). Indeed, all ghrelin-system components were significantly decreased in obese cort−/− compared with obese cort+/+ mice (Figure 1). When comparing the changes in the ghrelin-system at the pancreatic islet level with those observed at the whole pancreas, we observed a similar, albeit not identical, dysregulation of ghrelin system expression (Fig. 2). Specifically, ghrelin, and GHS-R transcript levels were also significantly higher in obese vs. lean cort+/+ mice, changes that were not observed in obese cort−/− mice. However, In1-ghrelin transcripts remained unchanged in the islet-enriched fraction of cort−/− obese mice, while its mRNA level significantly increased as a consequence of a HFD in cort −/− mice (Fig. 2).

### Modulation of Plasma Insulin Levels as Well as Insulin Expression in the Whole and Endocrine Pancreas Level in Obese Cort+/+ and Cort−/− Mice

**Figure 3:**
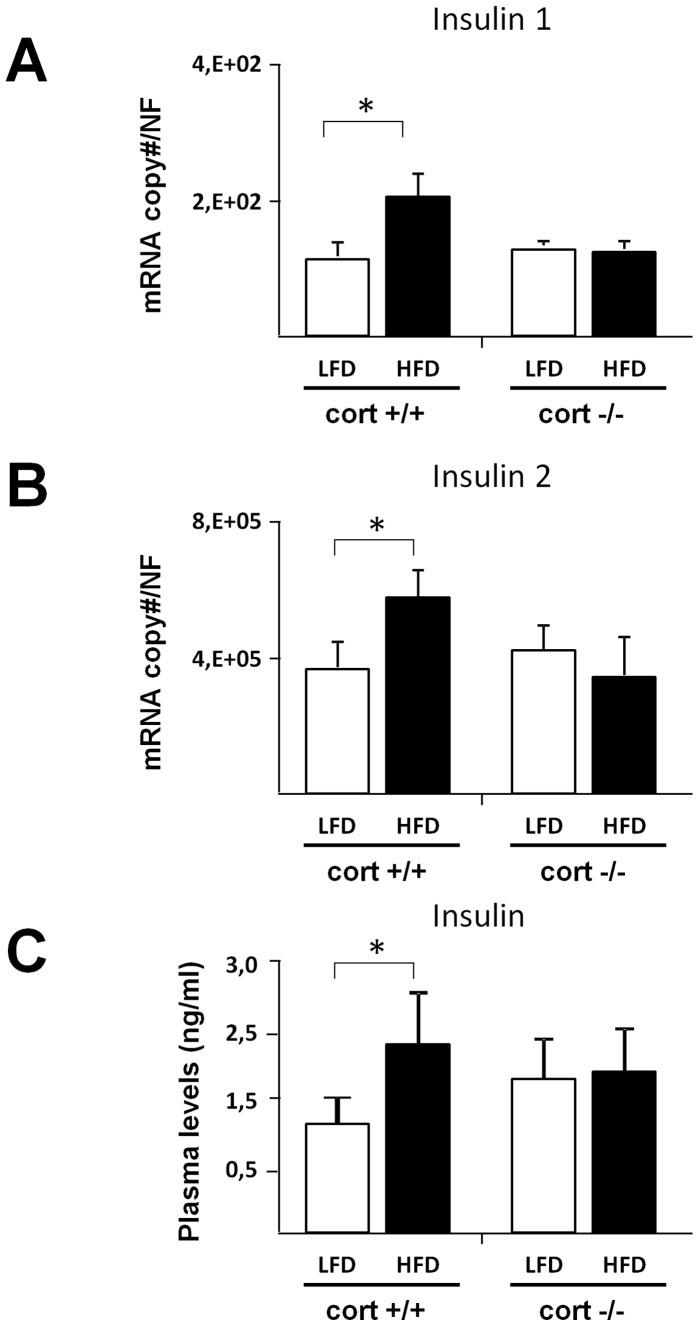
Expression profile of insulin 1 and insulin 2 (panels A and B, respectively) in the whole pancreas of cort+/+ and cort−/− male mice fed a low fat (LF) or high fat diet (HFD). C, plasma insulin level obtained from cort+/+ and cort−/− animals fed a LF- or HF-diet. Values represent mean ± SEM of copy number adjusted by normalization factor (NF) per 100 ng of cDNA obtained from 4–7 animals per experimental group. (*: p<0,05).

**Figure 4:**
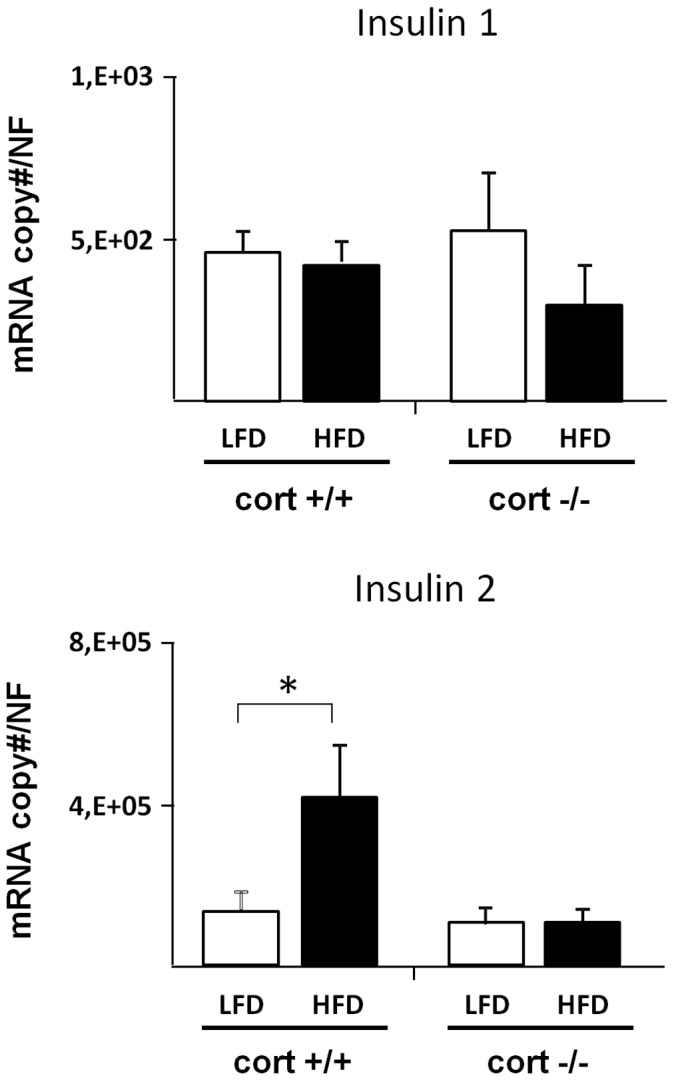
Expression profile of Insulin 1 and insulin 2 in pancreatic islets of cort+/+ and cort−/− male mice fed a low fat (LF) or high fat diet (HFD). Values represent mean ± SEM of copy number adjusted by β-actin expression per 100 ng of cDNA obtained from 2–3 animals per experimental group. (*: p<0,05).

Expression of insulin as well as plasma insulin levels were analyzed in cort+/+ and cort−/− mice under LFD and HFD conditions. As expected, insulin expression was significantly elevated in the whole pancreas (black columns in Fig. 3 A and B) of HFD-fed compared to LFD- fed cort+/+ mice; while at pancreatic islet level, only insulin-2 expression was significantly elevated (white columns in Fig. 4). Plasma insulin levels were also significantly elevated in cort +/+ mice under obesity conditions (white columns in Fig. 3C). Importantly, the increased levels of both transcript and plasma insulin as a consequence of an obese state were completely prevented in the absence of endogenous CORT (black columns in Fig. 3A, 3B and 4).

### Evaluation of in vitro CORT Action o Insulin Release

**Figure 5:**
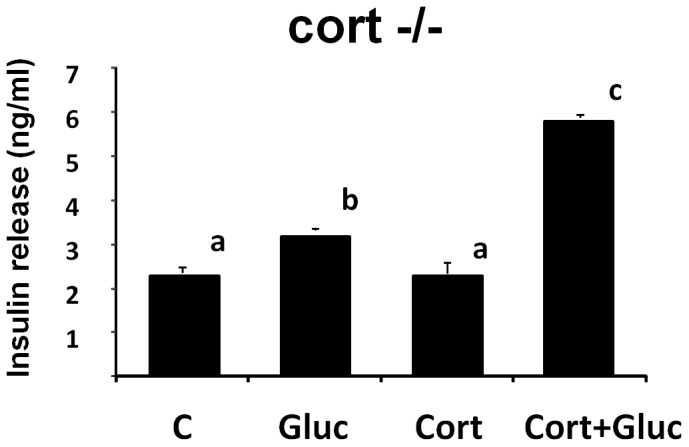
Evaluation of in vitro CORT action on glucose induced-Insulin release in cultured pancreatic islets from cort−/− mice. Values represent mean ± SEM obtained from 3 independent experiments. Different letters stand for statistical differences (p<0,05).

In order to evaluate the role of CORT on glucose-dependent insulin secretion effect,pancreatic islets from cort−/− mice were cultured and treated with 10–8 M CORT, 25 mM glucose or its joint administration, and then the insulin release was evaluated after 24 h treatment (Fig. 5). Our data reveal that single administration of CORT do not modify basal insulin release while combined administration of CORT and glucose enhances the stimulatory effect of glucose on insulin release (Fig. 5).

## Discussion

This study demonstrates that endogenous cortistatin markedly influences the expression pattern of various components of the pancreatic ghrelin system under both normal and obesity conditions. In addition, our data support the idea that cortistatin play important actions on pancreatic function, as the lack of cortistatin prevents the observed increase in the expression of insulin and its circulating plasma levels in obese mice.

Recently, several studies have described that the pancreas synthesizes all different components of ghrelin system, as reported in the beta, epsilon or another endocrine cells [5], [6], [8], [9], [10], [12], [21], [22], [23], [24], [25]. Accordingly, besides the well-documented regulation of pancreatic function by ghrelin from stomach origin [26], [27], [28], [29], it has been also proposed that locally produced ghrelin may have a role on pancreatic function and, specifically, on glucose homeostasis. Indeed, Dezaki *et al.* suggested that locally produced ghrelin regulates insulin production in a paracrine manner [30], although this aspect is still controversial [4] and consequently, a definitive role has not been defined yet. In the present study, we confirmed pancreatic expression of ghrelin system components (ghrelin, In1-ghrelin, GHS-R and GOAT) in the whole pancreas and islet-enriched cultures of control and cort−/− mice. In addition, a significant down-regulation of GOAT transcripts was observed in cort−/− mice, suggesting a possible regulation of ghrelin actions by cortistatin at pancreatic level. Similarly to that reported at the pituitary and hypothalamus [13], we also observed that pancreatic In1-ghrelin expression was significantly higher than that of native ghrelin, thus supporting the idea that this variant might be the predominant ghrelin gene derived-transcript at the pancreatic level, and also suggesting that it may exert important biological actions through a receptor still to be identified. Of note, In1-ghrelin variant retains the acylation site of pre-proghrelin [10] and, consequently, it may encode a peptide susceptible for acylation by GOAT action. Based on the observed down-regulation of GOAT expression in cort−/− mice, CORT might also regulate the putative pancreatic function of In1-ghrelin by regulating its acylation and consequently diminishing its local activity, an aspect that needs further attention.

It has been previously reported that ghrelin system components are regulated by certain metabolic conditions, like obesity, in the stomach, pituitary and hypothalamus [17], [19], [31]. In line with this, the present report is the first to reveal, to the best of our knowledge, that ghrelin system components are also markedly altered under DIO conditions at pancreatic level. However, contrary to that previously reported at the hypothalamic and pituitary level [17], [19], we observed an up-regulation of ghrelin, In1-ghrelin and GHS-R transcripts at the pancreas of cort+/+ (control) animals under obesity conditions, while GOAT expression level remains unaltered, thus supporting that obesity-induced alterations in the ghrelin-system are tissue-dependent. It is well known that obesity is a chronic inflammation condition that causes a deleterious effect on pancreatic function and, in particular, on beta-cell function [32]. In this sense, the observed increase in pancreatic ghrelin system expression may locally acts as a protective mechanism by promoting beta-cell survival and growth, a function that has been previously described for different ghrelin system components under physiological or pathological conditions [33], [34]. In addition, and similarly to that described for pancreatic ghrelin system profile, In1-ghrelin variant is also predominantly expressed under extreme metabolic conditions (obesity). These data again support a relevant role of this ghrelin variant at the pancreatic level that could be, at least in part, responsible for the described actions of ghrelin related peptides on beta-cell survival [33], [34].

In clear contrast to the up-regulation of ghrelin system under HFD conditions in cort +/+ mice described above, we observed a clear shut-down of ghrelin system components in the whole pancreas and islet-enriched culture of obese CORT deficient animals. These findings would support a novel pathophysiological role of CORT in the pancreatic function in individuals under metabolic stress (obesity). Conversely, at the endocrine pancreas, In1-ghrelin expression increased in cort−/− obese animals, throughout a mechanism still unclear that would specifically favor the splicing of ghrelin gene to generate In1-ghrelin variant. These findings suggest that native ghrelin and In-1 ghrelin variant might be differentially regulated in cultured pancreatic islets in a similar manner to that reported in the pituitary, hypothalamus and stomach [13], [19], [31].

Besides ghrelin expression, our data also reveal a significant increase of both insulin 1 and 2 mRNA transcripts in extracts from whole pancreas (only insulin 2 transcripts in islet samples) under obesity conditions. These data are in agreement with the elevation of plasma insulin levels in obese animals observed in this study and also documented by others [17], [33], [35]. However, in the pancreas of HFD-fed cort−/− animals, insulin expression remains at control (LFD-fed) levels, although significantly diminished in islet samples, thus suggesting and supporting a role for endogenous CORT in regulating endocrine pancreas function, particularly on beta-cell function.

### Conclusions

The present study provides empirical evidence for an increased pancreatic ghrelin system expression under obesity conditions. Moreover, our findings demonstrate that the lack of endogenous CORT (cort−/− mice) prevents the up-regulation of all components of the pancreatic ghrelin system as well as insulin secretion (mRNA expression and release) induced by obesity in cort+/+ mice, thereby suggesting that endogenous CORT may be a key regulator of pancreatic endocrine function under normal and particularly under pathophysiological states (obesity).
